# Neurophilin-1 in tumor growth

**DOI:** 10.18632/oncotarget.734

**Published:** 2012-11-05

**Authors:** Shailendra Kapoor

**Affiliations:** University of Illinois at Chicago, Chicago, IL

The recent article by Prud'homme et al [1] suggests that neuropilin-1 may contribute to tumor progression in a number of systemic malignancies and that it's down regulation may markedly decrease tumor growth. For instance, up regulation of neuropilin-1 levels results in a poor clinical outcome in tongue squamous cell carcinomas. Shorter overall survival is seen in oral tumors with NRP1/SEMA3A ratio greater than one [2]. Overall, invasiveness in these tumors is attenuated by knockdown of neuropilin-1. Neuropilin-1 knock- down is accompanied by attenuation of intra- tumoral vimentin levels [3]. A simultaneous increase in intra- tumoral E- cadherin levels is seen. Neuropilin-1 knockdown may be a potential therapeutic modality for treatment of tongue malignancies. Similarly, increased expression of neuropilin-1 is seen in breast carcinoma stem like cells. This accentuation of neuropilin-1 levels results in altered NF-κB activation and subsequent formation of tumor mammospheres [4]. Mammosphere formation is significantly attenuated by knock- down of neuropilin-1 by siRNA. Neuropilin-1 knock- down also results in decreased ERK1/2 phosphorylation. Thus neuropilin-1 may be a potential target for abrogation of tumor growth in breast carcinomas. Similarly, increased neuropilin-1 levels are seen in colorectal carcinomas. Agents such as miR-320a abrogate the risk of developing hepatic metastasis in these tumors [5]. miR-320a mediates this effect by down- regulating neuropilin-1 expression and the near future may see its increased use to attenuate the risk of liver metastasis from colorectal malignancies. Increased neuropilin-1 expression is also seen in pancreatic malignancies. The administration of sema3A-lytic hybrid peptide abrogates growth and progression in pancreatic malignancies by binding to neuropilin-1 [6]. Neuropilin-1 also promotes tumor growth in hepatocellular carcinomas by modulating vascular remodeling [7].

The above examples clearly illustrate the role of neuropilin-1 in tumor progression in systemic malignancies and the urgent need to identify further inhibitors of neuropilin-1 expression.
